# Improvement of 5-fluorouracil chemosensitivity in colorectal cancer cells by siRNA-mediated silencing of STAT6 oncogene

**DOI:** 10.22038/IJBMS.2023.74275.16136

**Published:** 2024

**Authors:** Omid Rahbar Farzam, Behzad Baradaran, Bahman Akbari, Souzan Najafi, Mohammad Amini, AmirHossein Yari, Reza Dabbaghipour, Vahid Pourabdollah Kaleybar, Shiva Ahdi Khosroshahi

**Affiliations:** 1 Department of Medical Biotechnology, School of Medicine, Kermanshah University of Medical Sciences, Kermanshah, Iran; 2 Immunology Research Center, Tabriz University of Medical Sciences, Tabriz, Iran; 3 Medical Biology Research Center, Kermanshah University of Medical Sciences, Kermanshah, Iran; 4 Department of Biology, Tabriz Branch, Islamic Azad University, Tabriz, Iran; 5 Medical School, Shiraz University of Medical Sciences, Shiraz, Iran

**Keywords:** 5-Fluorouracil, Chemosensitivity, Colorectal cancer, siRNA, STAT6

## Abstract

**Objective(s):**

Colorectal cancer (CRC) remains a major health concern worldwide due to its high incidence, mortality rate, and resistance to conventional treatments. The discovery of new targets for cancer therapy is essential to improve the survival of CRC patients. Here, this study aims to present a finding that identifies the STAT6 oncogene as a potent therapeutic target for CRC.

**Materials and Methods:**

HT-29 CRC cells were transfected with STAT6 siRNA and treated with 5-fluorouracil (5-FU) alone and combined. Then, to evaluate cellular proliferation and apoptosis percentage, MTT assay and annexin V/PI staining were carried out, respectively. Moreover, the migration ability of HT-29 cells was followed using a wound-healing assay, and a colony formation assay was performed to explore cell stemness features. Gene expression was quantified via qRT-PCR. Afterward, functional enrichment analysis was used to learn in-depth about the STAT6 co-expressed genes and the pathways to which they belong.

**Results:**

Our study shows that silencing STAT6 with small interfering RNA (siRNA) enhances the chemosensitivity of CRC cells to 5-FU, a commonly used chemotherapy drug, by inducing apoptosis, reducing proliferation, and inhibiting metastasis. These results suggest that combining 5-FU with STAT6-siRNA could provide a promising strategy for CRC treatment.

**Conclusion:**

Our study sheds light on the potential of STAT6 as a druggable target for CRC cancers, the findings offer hope for more effective treatments for CRC patients, especially those with advanced stages that are resistant to conventional therapies.

## Introduction

Colorectal cancer (CRC) is the most frequent form of the disease, and it may affect either the rectum or the colon. When it comes to cancer-related mortality, colorectal disease is a close second (1, 2). CRC is caused by aging, bad lifestyle choices, genetic changes, and persistent intestinal inflammation (3). Regrettably, around fifty percent of individuals with CRC are diagnosed at late stages when they are no longer amenable to effective treatment with conventional methods such as surgery, radiation, and chemotherapy (4). The development of resistance to adjuvant medicines such as chemotherapy and radiation is another factor that contributes to the high death rate associated with CRC (5). As a consequence, the development of new therapeutic methods for the treatment of CRC necessitates the discovery of new molecular pathways that are responsible for metastasis, carcinogenesis, and drug resistance in CRC (6).

Patients diagnosed with CRC are often given 5-fluorouracil (5-FU) to prevent metastasis and recurrence of the disease; however, chemoresistance and unpleasant side effects have limited its administration (7). Small interfering RNA therapy, also known as siRNA therapy, promotes the development of treatment procedures that are therapeutically relevant and successful. This helps researchers overcome the obstacles they face. Targeting mRNA expression and reducing the expression of cancer-causing genes may be accomplished via the use of RNA interference (RNAi)-based therapy modalities in the treatment of cancer (8). 

Differentiation, apoptosis, angiogenesis, and proliferation are all regulated by STAT transcription factors (8). There are seven STAT genes in the human genome, and all of them are responsible for a variety of effects caused by the extracellular signaling pathway. These effects are mostly caused by the STAT genes’ ability to change gene transcription in effector cells. Members of the STAT family, in particular STAT6, are shown to be associated with the initiation, metastasis, progression, survival, and treatment resistance of human cancer. Under normal conditions, two cytokines, namely interleukin-13 and interleukin-4, work together to activate the transcription factor STAT6 (9).

The JAK/STAT6 pathway, also known as the Janus kinase/STAT6 pathway, is activated by interleukin-13 and interleukin-4. The subsequent cellular response to stimulation by IL-13 and IL-4 is determined by STAT6, which acts as a mediator in the signal transduction process. On the other hand, IL-13 and IL-4 produced aberrant activation of STAT6, which boosts pro-metastatic activity in cancer cells such as invasion, migration, survival, and proliferation (10).

Recent studies investigated the potential effects of 5-FU therapy combined with STAT6-siRNA on cells from patients with CRC. In order to induce apoptosis, inhibit the proliferation and migration of CRC cells, and provide hope for patients, this research examines the synergistic effects of decreasing STAT6 gene expression using siRNA and the 5-FU drug. In order to achieve this goal, siRNA was transfected into CRC cells. Following this, the cells were treated with 5-FU, and a variety of cellular and molecular techniques were utilized to learn a great deal regarding the function of this transcription factor in the progression of CRC.

## Materials and Methods

***Cell culture***

HT-29, SW480, and HCT116 cell lines came from the cell bank located at the Pasture Institute in Tehran, Iran. Cells were grown in a medium consisting of RPMI-1640 that had 10% fetal bovine serum (FBS) and 1% penicillin and streptomycin added to it (Gibco, Gaithersburg, MD, USA). The cells were kept in an incubator at 37 °C with 5% carbon dioxide and 95% humidity as recommended by the standard protocols for cell culture. Trypsin (Trypsin-EDTA 0.25%, Gibco, USA) was used to detach the cells after reaching about 70 to 80% confluence in the log growth phase.

***siRNA transfection***

STAT6-siRNA at various concentrations (60, 80, and 100 pmol) were transfected into HT-29 cells (5×10^5^cells/ml) using Gene Pulser Xcell Electroporation System (Bio-Rad, Hercules, California, USA) based on the protocol provided by the manufacturer (cuvettes: 0.4 cm^3^, capacitance 500, resistance ∞ and voltage: 170 v). The cells at the density of 5 × 10^5^ cells/well were seeded into 6-well plates and incubated for various periods, including 24, 48, and 72 hr. The quantitative reverse transcription-PCR (qRT-PCR7) analysis was used to evaluate the outcome of STAT6-siRNA transfer and additionally to determine the optimum dose and time of STAT6-siRNA for further experiments. The confirmed sequence of sense and antisense STAT6-siRNA strands is shown in Table 1.

***qRT-PCR analysis***

The reduction in STAT6 expression at the mRNA level in the HT-29 cell line was demonstrated by qRT-PCR. To get total RNA from the cells, the Trizol RNA extraction kit was applied in the manner described in the protocol (GeneAll, Korea). To determine the level of purity and concentration of the extracted RNA, a NanoDrop2000 spectrophotometer was utilized (Thermo Scientific, Waltham, MA, USA). Pure RNA was converted into cDNA by using a thermal cycler system manufactured by Bio-Rad and a cDNA synthesis kit manufactured by BIOFACT (Republic of Korea). qRT-PCR was carried out with the SYBR Premix Ex Taq (Takara Bio, Otsu, Shizuoka, Japan) using the following cycling criteria in a light cycler system (Roche Diagnostics, Mannheim, Germany) to investigate the relative expression levels of *Bax, Bcl2, Caspase9, CDK4, Cyclin D1, MMP9, CD44, Sox2 *genes: initial denaturation at 95 °C (15 min), 45 cycles of 95 °C (10 sec), and 60 °C (1 min). In a total volume of 20 μl, each reaction contained 4 pmol of forward and reverse primers, 8 μl of ddH_2_O, 1000 ng of cDNA, and 10 μl of SYBR® Green real-time PCR master mix. The GAPDH gene was employed as a housekeeping gene. Table 2 contains a listing of the primer sequences that were utilized in this experiment. 

***Cell viability assay (MTT assay)***

The 3- [4, 5-dimethylthiazol-2-yl]-2,5 diphenyl tetrazolium bromide (MTT) assay (MTT assay kit, Sigma) was used to evaluate the cytotoxicity of STAT6-siRNA and IC50 of 5-FU on HT29 cells. Two different cultures of HT-29 cells (transfected and non-transfected) were seeded in 96-well plates and treated with 0.01 μg/ml–100 μg/ml of 5-FU (injection, 50 mg/ml, EBEWE). 50 μl MTT solution (2 mg/ml) was added to the wells 48 hr after transfection and 24 hr after treatment and then incubated. After 4 hr, the medium of each well was changed, and 150 μl dimethyl sulfoxide (DMSO) was added. The plate was incubated for 15 min and then shaken for 10 min in a dark situation to dissolve formazan crystals. Then, the optical density (OD) of each well was measured at 570 nm wavelength with an ELISA reader (Sunrise RC, Tecan, Switzerland).

***Apoptosis assay***

The annexin V/propidium iodide (PI) test was utilized in order to evaluate the rate of apoptosis induction that was brought about by the combination of STAT6-siRNA transfection and 5-FU treatment. Apoptosis-mediated cell death was examined by using a V-FITC/PI staining kit (Roche, Germany) and staining the cells in accordance with the instructions provided by the manufacturer. In the beginning, 5 x 10^5^ HT-29 cells were seeded into each well of a 6-well plate and allowed to grow in culture for 24 hr. After being exposed to the various treatments, the cells were analyzed and assigned to one of four groups: control, STAT6-siRNA, 5-FU, or combination. After the cells were detached, they were centrifuged at 1300 rpm for ten min at a temperature of 4 °C. After adding binding buffer (200 ul), annexin V (5 ul), and PI (5 ul) to the samples to label them, the samples were placed back into the refrigerator and allowed to incubate in the dark for 15 min. In order to evaluate the labeled cells, flow cytometry (MiltenyBiotecTM FACS Quant10; MiltenyBiotec, Germany) was utilized, and the analysis of the data obtained was performed with the help of the FlowJO program (FlowJo LLC., Ashland, OR, USA). 

***Cell cycle assay***

To investigate the impact that STAT6-siRNA and 5-FU have on the progression of the cell cycle, flow cytometry was used. A 6-well plate containing both transfected and untransfected cells was seeded with a density of 5 x 10^5^ cells in each well, and the plate was then placed in an incubator for 24 hr. Following the treatment step with the drug, the cells were detached using trypsin and subsequently washed with PBS. After being centrifuged at 1300 rpm for ten min, the cells were preserved in ice-cold ethanol at a temperature of -20 °C for a whole night. After adding the samples to a solution that included 1 mg/mL RNase A (Bioneer, Daejeon), the mixture was then heated to 37 °C for half an hour. After that, the samples were incubated for ten min at room temperature in the dark in a DAPI solution that contained 0.1% DAPI (1 mg/ml) and 0.1% Triton X100 in PBS. The sub-G1, G0-G1, S, and G2 stages of the cell cycle of the stained cells were assessed with the assistance of a MACSQuant flow cytometer. To analyze the gathered information, the FlowJo software was used (Tree Star, San Carlos, CA, USA).

***Wound healing assay***

The wound-healing assay was carried out to investigate the impact of STAT6-siRNA, 5-FU, and the combination on the capacity of HT-29 cells to migrate. Following the transfection, the cells were seeded into 24-well plates at a density of 1×10^6^ cells per well as specified in the protocol. Before being treated with 5-FU, the tip of a yellow pipette was used to make a small scratch. The photographs of cells migrating to the wound area were taken at 0 hr, 24 hr, and 48 hr after scratch via the inverted microscope (Optika, Bergamo, Italy).

***Colony formation assay***

An experiment involving the creation of colonies was carried out to investigate the clonogenic potential of HT-29 cells. The density of 1 × 10^3^ transfected and non-transfected cells was incubated in each well at 37 °C for ten days. After being cleaned with PBS, the cells were stained with a crystal violet staining dye containing 0.5% crystal violet, formaldehyde, and methanol. After incubating the wells for forty min, the colonies were counted using the image processing program Image.

***Functional enrichment analysis***

To determine STAT6’s molecular functions and associated pathways, STAT6 co-expression genes were extracted using the GEPIA online tool (11). Next, a pathway enrichment analysis was conducted on these genes. To determine which biological pathways these genes influence, the Kyoto Encyclopedia of Genes and Genomes (KEGG) database was queried with the web application Enrichr (12-14) and performed a pathway enrichment analysis. The Cytoscape program was then utilized to visualize the results.

***Statistical analysis***

The results of each trial were reported as the mean value along with the standard deviation (SD) in triplicate. t-test and one-way analysis of variance were used so that the statistical analysis could examine the differences between the groups. In order to conduct the statistical analysis, the GraphPad Prism software, version 8.0, was utilized. To establish statistical significance, a *P*-value that was lower than 0.05 was required.

## Results

***Evaluation of STAT6 expression in colorectal cancer cell lines***

**Figure 1:**
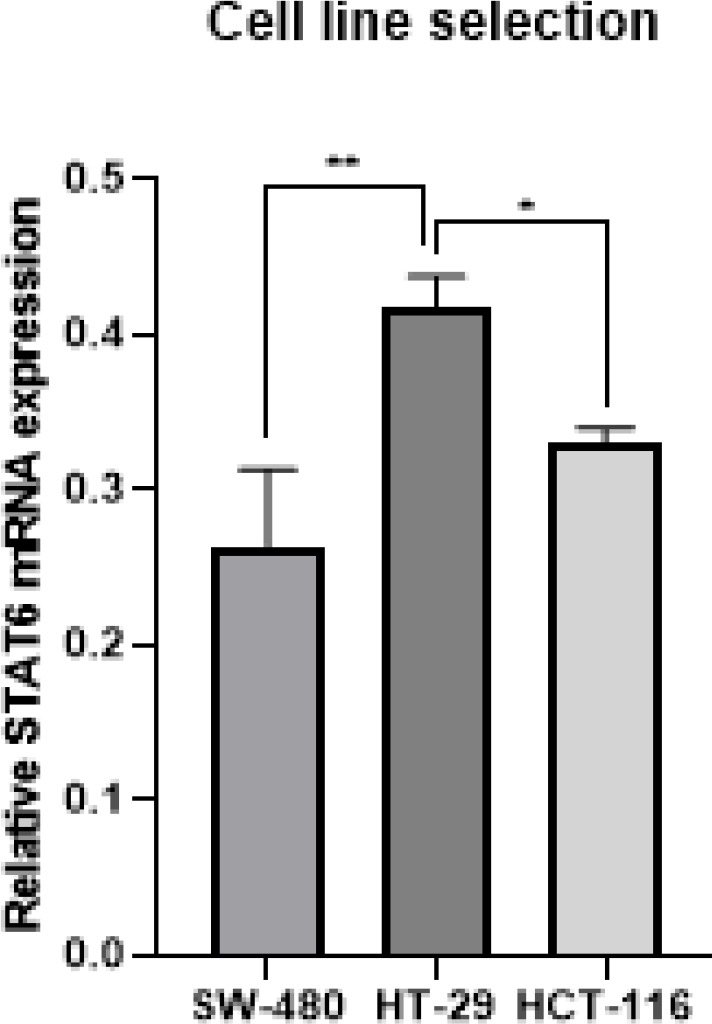
Investigation of STAT6 expression in the CRC cell lines

The expression of STAT6 in CRC cell lines, namely HCT116, HT-29, and SW480, was analyzed using qRT-PCR. When compared to other cell lines, the HT-29 cell line had a significantly higher level of STAT6 expression (Figure 1). As a direct consequence of this finding, the HT-29 cell line was selected for further investigation.

***Decreased STAT6 mRNA levels after STAT6-siRNA transfection***

**Figure 2:**
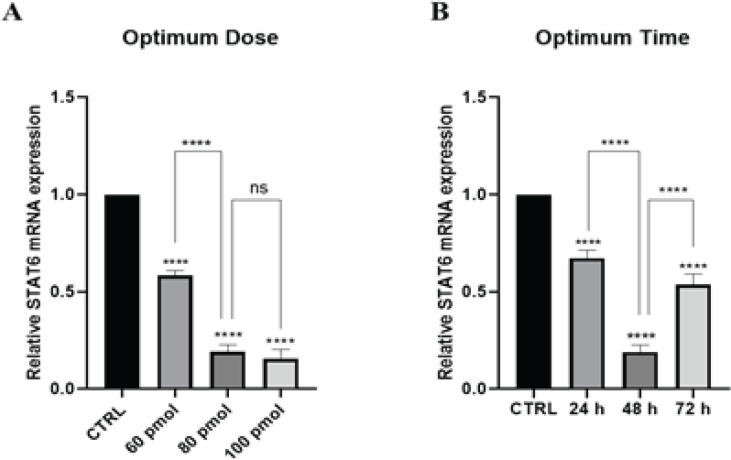
Impact of STAT6 siRNA at various periods and dosages on STAT6 expression in HT-29 cell line

HT-29 cells were transfected with siRNA at 60, 80, and 100 pmol, and qRT-PCR was used to weigh the effectiveness of STAT6-siRNA suppression. The proposed siRNA could knock down STAT6 expression in a dose-dependent manner, the finding is given in (Figure 2A). Forty-eight hours after transfection with the optimum dose, STAT6-siRNA was found to be significantly suppressed (Figure 2B). Following the findings of this study, cell transfection with 100 pmol siRNA was considered the most effective concentration of transfection for up to 48 hr.

***Down-regulation of STAT6 increased the HT-29 cells’ sensitivity to 5-FU***

**Figure 3.**
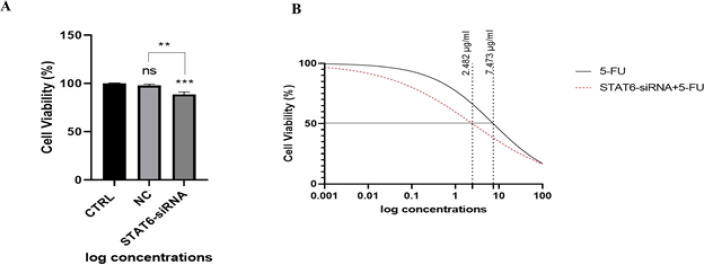
Effect of STAT6-siRNA separate and in combination with 5-FU on cell proliferation in HT-29 cell line

MTT test was used to determine the effect of STAT6-siRNA on cell viability and whether STAT6 suppression may improve the sensitivity of HT-29 cells to 5-FU. According to the results, in comparison with the control group, STAT6-siRNA transfection significantly reduced cell survival rates in HT-29 cells (*P*<0.001) (Figure 3A). Furthermore, STAT6 silencing combined with 5-FU indicated a constant reduction in HT-29 cells’ viability and increased HT-29 cells’ sensitivity to 5-FU. When comparing the combined and separate groups, the 5-FU IC_50_ was decreased from 7.473 ug/ml to 2.482 ug/ml, revealing that STAT6 can play an important role in the resistance of colorectal cancer cells to 5-FU (Figure 3B).

***Apoptosis induction in HT-29 cells by combining STAT6-siRNA with 5-FU***

**Figure 4:**
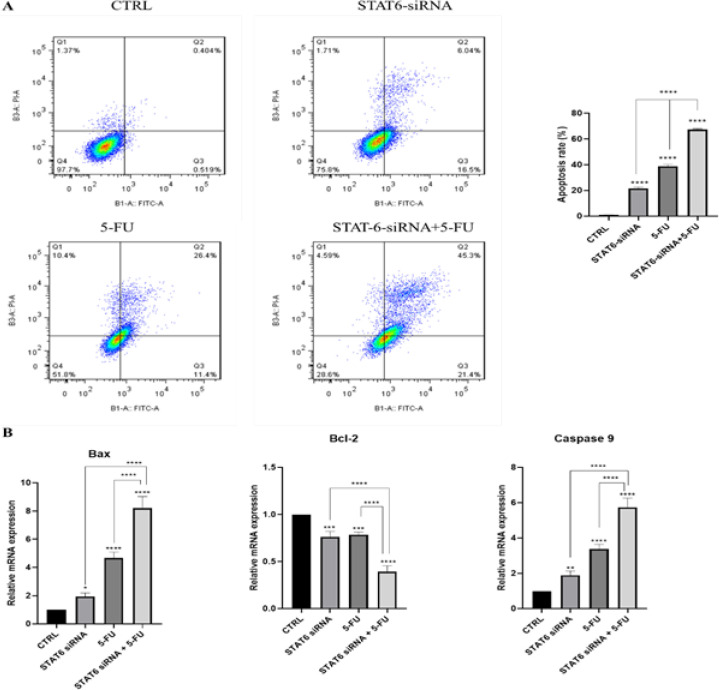
A) Annexin V-FITC/PI test revealed that STAT6-siRNA-transfected cells that were treated with 5-FU had higher apoptotic activity than STAT6-siRNA-transfected cells with no 5-FU treatment. B) The combined group qRT-PCR results showed a significantly higher expression of caspase9 and Bax but a significantly lower expression of Bcl2 (*P*<0.05, *P*< 0.01, *P*<0 .001, *P*<0.0001).

The annexin V/PI assay was performed on HT-29 cells to determine whether or not STAT6-siRNA alone or in combination with 5-FU might influence the apoptotic death of the cells. According to the findings obtained, decreased STAT6 expression considerably facilitated the induction of apoptosis by 5-FU in HT-29 cells. The apoptosis rates in HT-29 cells for STAT6-siRNA and 5-FU were 22.54% and 37.8%, respectively. Interestingly the apoptosis rate in the stat6-siRNA/5-FU combination group was 66.7% which was much higher when compared with other groups (Figure 4A). Furthermore, qRT-PCR was used to examine the expression of apoptosis genes implicated in the molecular process of apoptosis. Compared with independent groups, down-regulation of STAT6 in combination with 5-FU enhanced *Bax* gene expression and reduced *Bcl2* gene expression. Furthermore, when simultaneous STAT6 suppression with 5-FU was compared with the control, significant alterations in *caspase9* expression were observed. (Figure 4B). HT-29 cells were sensitized to 5-FU via apoptosis induction following co-treatment with STAT6-siRNA and 5-FU. This suggests that STAT6 plays a crucial role in 5-FU sensitization.

***Arrested the cell cycle in the sub-G1 phase by combining STAT6-siRNA with 5-FU***

**Figure 5:**
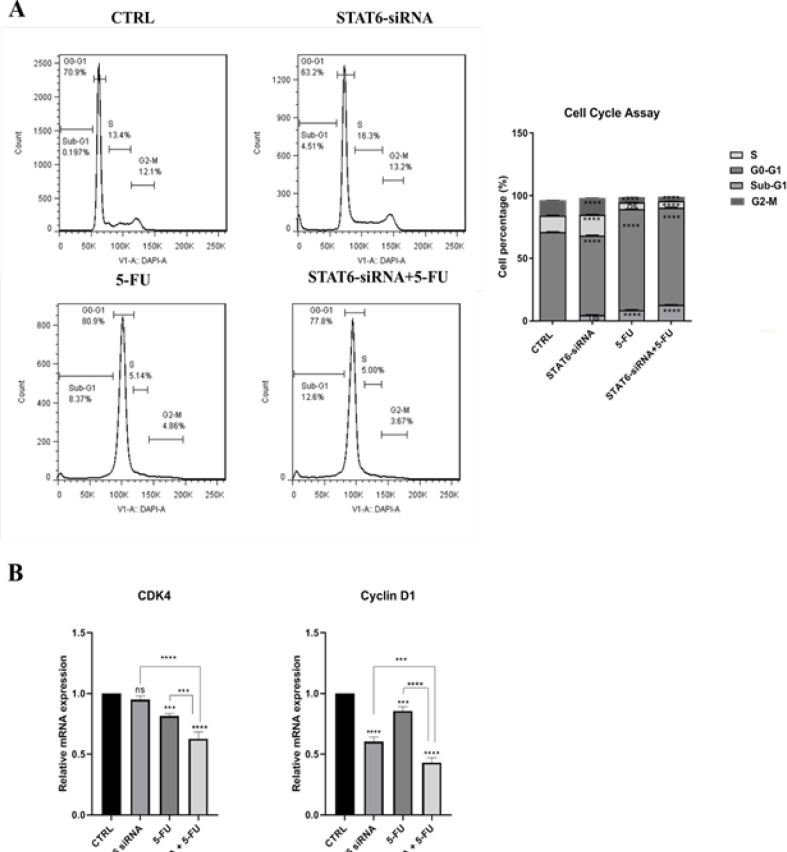
Effects of STAT6-siRNA, 5-FU treatment and combined therapy on HT-29 cell cycle distribution

The combination therapy effects of STAT6-siRNA and 5-FU on cell cycle distribution in HT-29 cells were investigated using flow cytometry. In the combination treatment, the percentages of cells in the sub-G1 phase were much higher than when STAT6-siRNA and 5-FU were given separately. The findings showed a considerable rise in the sub-G1 phase in different groups, with increases of 4.51% and 8.37% in the STAT6 knockdown and 5-FU treatment groups, respectively. The combination treatment resulted in a more significant sub-G1 population of 12.6%, indicating that the combined group significantly promoted cell death in HT-29 cells (Figure 5A). To confirm cell cycle results, the expression of *CDK4* and *cyclin D1* was explored by qRT-PCR (Figure 5B). According to the findings, in the combined group, the expression of *CDK4* and *cyclin D1* were significantly decreased compared to single groups (*P*<0.0001).

***Combining STAT6-siRNA and 5-FU decreased the migration of HT-29 cells***

**Figure 6:**
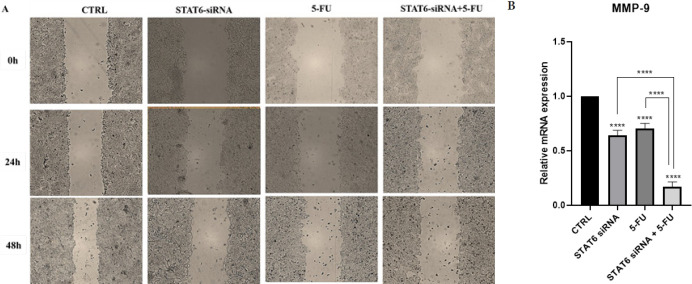
Effects of STAT6-siRNA, 5-FU treatment and combined therapy on HT-29 cell migration

The wound-healing test was used to investigate whether STAT6 knockdown and 5-FU treatment could affect the migration of HT-29 cells. STAT6-siRNA and 5-FU reduced cell mobility in wound spaces based on the rate of migrating cells. The combined treatment considerably decreased the migration of tumoral cells compared to single groups, suggesting that STAT6 could show a critical role in HT-29 cells’ capacity to migrate (Figure 6A). Following the molecular evaluation, compared with those that received either therapy alone, the combination STAT6-siRNA and 5-FU treatment resulted in a significant reduction in the relative expression of *MMP9* (*P*<0.0001) (Figure 6B).

***Combination treatment inhibited the clonogenicity of HT-29 cells***

**Figure 7:**
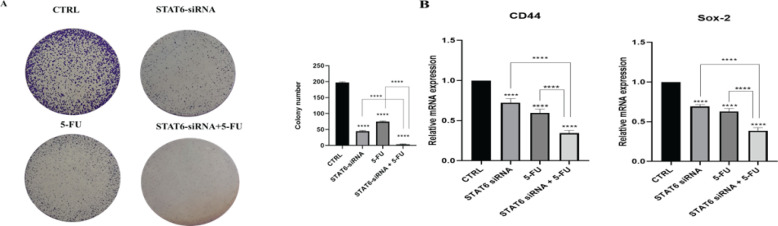
STAT6-siRNA suppression effect combined with 5-FU treatment on colony formation of HT-29 cells

Using a colony-forming assay, the impact of coupled STAT6-siRNA and 5-FU on the stemness characteristics of HT-29 cells was examined. As observed in (Figure 7A), while STAT6 knockdown and 5-FU therapy alone reduced the colony formation rate, the combined group formed fewer colonies. To investigate the molecular pathways involved in stemness, the expression levels of *CD44* and *Sox2* were also measured using qRT-PCR. *Sox2* and *CD44* expression levels were dramatically decreased by 5-FU therapy with STAT6 suppression (*P*<0.0001) (Figure 7B). 

***STAT6 serves a crucial function in numerous pathways***

**Figure 8:**
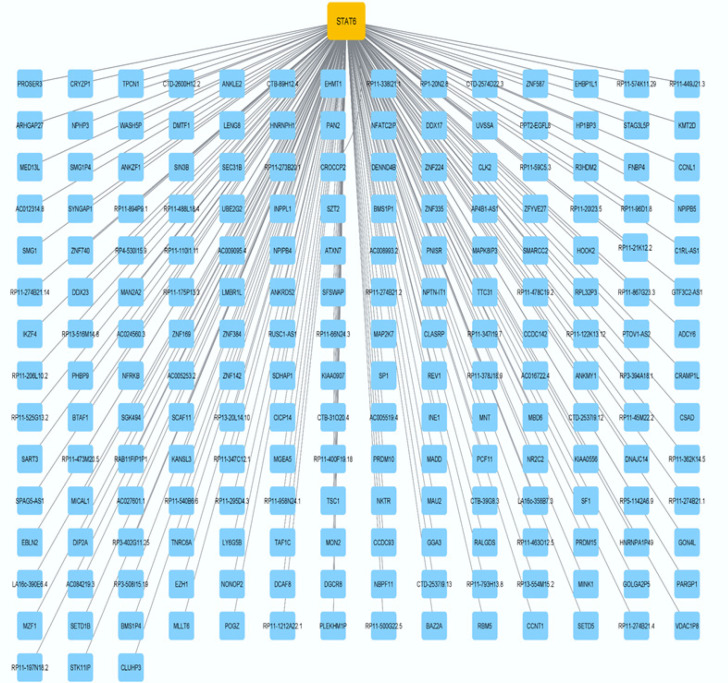
The genes that are co-expressed with STAT6

**Figure 9:**
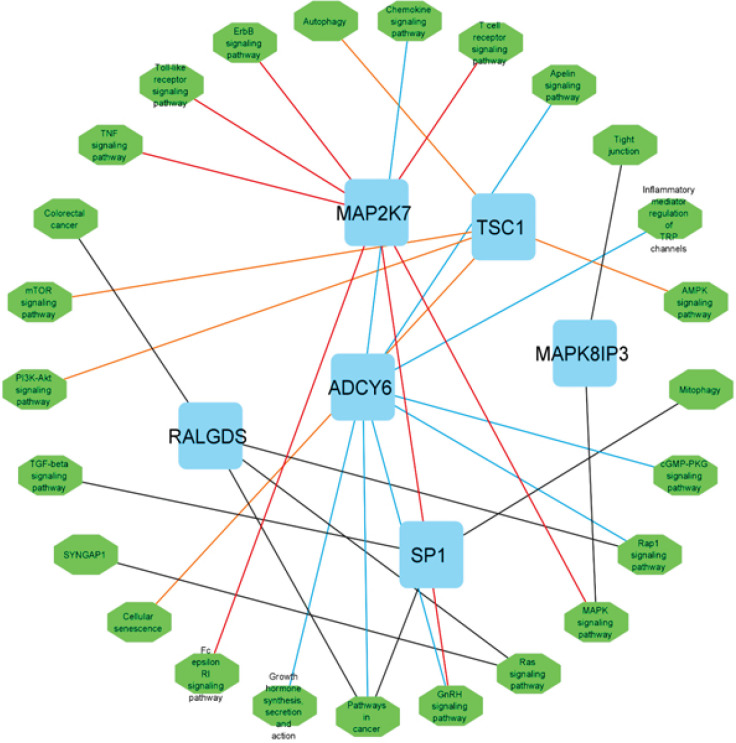
The genes essential involvement in the various pathways and displaying the pathways associated with the genes that are co-expressed

By utilizing the online tools provided by Gepia, we were able to query the TCGA database and learn which genes are co-expressed with STAT6. As a result, genes whose expression is associated with STAT6 were identified in CRC. (Figure 8) depicts the network of co-expression. The Enrichr online program was then used to query the KEGG pathway database, confirming the genes’ essential involvement in the various pathways (Figure 9) and displaying the pathways associated with the genes that are co-expressed.

**Table 1:** STAT6-siRNA sequence. STAT6 suppresion increases the sensitivity of CRC cells to 5-FU

**siRNA**	**Sense strand**	**Antisense Strand**
STAT6	5′-GACAUGUGGUUACUAGUACAGGUTT-3′	5′-AAACCUGUACUAGUAACCACAUGUCCA-3′

**Table 2:** Seqences of primers to investigate genes involved in apoptosis, cell cycle, migration, clonogenicity by real-time PCR

**Target**	**Forward primer (5′-3′)**	**Reverse primer (5′-3′)**
***Bax***	TTTGCTTCAGGGTTTCATCCA	TCTGCAGCTCCATGTTACTGTC
***Bcl2***	GAGTTCGGTGGGGTCATGTG	CACCTACCCAGCCTCCGTTA
***Caspase9***	CCTTCCTCTCTTCATCTCCTGCT	TTGCTGTGAGTCCCATTGGT
***CDK4***	CCATCAGCACAGTTCGTGAGGT	TCAGTTCGGGATGTGGCACAGA
***Cyclin D1***	TCTACACCGACAACTCCATCCG	TCTGGCATTTTGGAGAGGAAGTG
***MMP9***	ATTCATCTTCCAAGGCCAATCC	CTTGTCGCTGTCAAAGTTCG
***CD44***	CAGCCTACTGGAGATCAGGATGA	GGAGTCCTTGGATGAGTCTCGA
***SOX2***	ACATGTGAGGGCCGGACAGC	TTGCGTGAGTGTGGATGGGATTGG
***GAPDH***	AACATCATCCCTGCCTCTAC	CTGCTTCACCACCTTCTTG

The HT-29 cell line has a higher level of STAT6 expression than other cell lines (*P*<0.05; *P*<0.01), CRC: Colorectal cancer

A) 100 pmol of siRNA sinnificantly down-regulated STAT6 expression as compared to the control group and other dosages examined. B) 48 hr was selected as an optimum time (ns=not significant; *P*<0.0001)

A) STAT6 silencing reduced HT-29 cell proliferation compared with the control. B) In HT-29 cells, STAT6-siRNA silencing enhances chemosensitivity to 5-FU in a dosedependent manner. The data were shown as mean±SD (ns= not significant; *P*< 0.01; *P*<0.001).

A) Flow cytometry revealed a significant sub-G1 phase population in the combination group. B) In the combined group, CDK4 and cyclin D1 expression were significantly decreased by qRT-PCR. The findings were given as the mean of the experiments’ standard deviations (ns= not significant; *P*<0 .001, *P*< 0.0001). 

A) The rate of HT-29 cells migrated to the wound area showed that STAT6-siRNA suppression combined with 5-FU therapy reduced HT-29 cell migration. B) In the combination group, the results of qRT-PCR demonstrated a substantial decrease in the mRNA expression levels of MMP9 (*P*<0.0001).

A) Compared with single groups, the combined treatment had fewer colonies. B) After inhibiting STAT6-siRNA with 5-FU, qRT-PCR revealed a significant reduction in Sox2 and CD44 mRNA expression (*P*< 0.0001).

## Discussion

STAT6 serves as a potential oncogene causing a variety of human cancers, including CRC, breast cancer, and leukemia by modulating multiple signaling pathways (15, 16). CRC remains one of the deadliest human diseases due to distant metastases and chemoresistance development; therefore, new treatment development is needed to effectively treat CRC patients (17). Hence, this investigation aimed to see how STAT6 suppression affects chemosensitivity and cell migration in CRC cells when treated with 5-FU. STAT6 expression in HT-29 cells was reduced using STAT6-specific siRNA, and functional evaluations were conducted. MTT experiment revealed that STAT6 inhibition alone decreases the viability of HT-29 cells compared to controls. Furthermore, combination therapy reduced 5-FU IC50 from 7.473 ug/ml to 2.482 ug/ml, indicating that STAT6 silencing could raise the chemosensitivity of CRC cells to 5-FU treatment in this cell line. In line with our results, STAT6 participation in malignant cell drug responsivity to several chemotherapeutic drugs, such as oxaliplatin and Avastin has been documented (18).

STAT6 suppression using the RNA interference technique could inhibit proliferation through apoptosis induction in human HT-29 CRC cells (19). Consistently, our results also demonstrated that STAT6 suppression could induce apoptosis in HT-29 cells. As a result, STAT6 may have a role in activating apoptosis in CRC cells in response to 5-FU chemotherapy. Furthermore, flow cytometry analysis revealed that STAT6 inhibition, either alone or in combination with 5-FU, resulted in HT-29 accumulation in the sub-G1 phase, further confirming the anti-apoptotic role of STAT6 in CRC cells. In the current study, qRT-PCR was used to examine the mRNA expression levels of the main regulators of apoptosis, including *caspase9*, *Bax*, and *Bcl2*. In CRC cells, *STAT6* knockdown and 5-FU treatment promoted mitochondrial cytochrome C release, suppressed *Bcl2* expression, and up-regulated *Bax* and *caspase9* expression as a proapoptotic regulator. Along with our result, inhibiting STAT6 expression reduced *Bcl2 *expression in SW480 and HCT116 cells, resulting in apoptosis induction, according to Jiang and colleagues (20). As a result of our findings and previous investigations, the *Bax*/*Bcl2* ratio was modulated, and apoptosis was caused by STAT6 inhibition, making CRC cells more sensitive to 5-FU.

Metastasis, as a consequence of epithelial mechanical transition (EMT), is one of the primary contributors to the high mortality rate associated with CRC (21). EMT is a multi-step process that allows epithelial cells to acquire mesenchymal features. This gives epithelial cells the ability to spread and infiltrate other tissues (22). In light of this, the current study set out to determine whether or not STAT6 plays a role in the progression of metastatic characteristics seen in CRC. In the wound-healing assay, inhibiting STAT6, either on its own or in combination with treatment with 5-FU, significantly reduced the ability of HT-29 cells to migrate. These findings demonstrated the existence of a possible cooperative impact between inhibition of STAT6 expression and treatment with 5-FU on the reduction of CRC cell metastasis. It has been proven by Guo and colleagues that IL-13 causes EMT by elevating the ZEB-1 factor. They further demonstrated that inhibiting STAT6 leads to a reduction in the IL-13/STAT6 signaling pathway, which, in turn, results in a lower likelihood of EMT (23).

Additionally, to validate the anti-migratory effect that combination therapy had on HT-29 cells that were assigned to treatment groups, the expression level of *MMP9* was evaluated. According to the findings of qRT-PCR, inhibiting STAT6 with siRNA, either on its own or in combination with 5-FU treatment, resulted in a significant reduction in *MMP9* expression. MMPs are known to be essential in tumor invasion and metastasis (24). *MMP9* can induce metastasis, and its increased expression facilitates cancer metastasis (25). Further, in line with our findings, adjuvant medicines were shown to significantly slow tumor growth when used in combination with 5-FU, which was linked to a reduction in STAT6-phosphorylation. Lowering *MMP9* boosted the impact of 5-FU (26). Therefore, STAT6 can be identified as a key regulator of cancer cell migration, including CRC cell migration.

Besides, in various malignancies, STAT6 function has been demonstrated to be critical in preserving the stemness properties of cancer cells (27). In the current work, the stemness of HT-29 cells was studied to see what effect the STAT6 siRNA-mediated suppression might have. When compared to individual treatments and the control, the colony formation test revealed that STAT6 suppression with 5-FU therapy may dramatically diminish the number of colonies as well as their growth. Moreover, the expression levels of *CD44* and *Sox2* were examined to demonstrate the probable processes that influence the stemness features of CRC cells (28). In several malignancies, the *CD44* gene has been known as a crucial stemness-related factor (29). Also, *Sox2* is important in maintaining the self-renewal and stemness of cancer cells (30). In line with these results, increased *Sox2* expression was seen in round-shaped cells found in poorly differentiated regions, metastatic lesions, and the invasive frontier. In CRC cells, knockdown *Sox2* decreased the number of round-shaped cells and inhibited invasion, cell migration, tumorigenicity, and colony-forming potentiality (31). Also, combination therapy down-regulated *CD44* and *Sox2* genes in HT-29 cells. Therefore, suppressing STAT6 reduces the stemness properties of CRC cells via down-regulating stemness-related genes, i.e., *CD44* and *Sox2* expression. 

STAT6 is a pivotal gene that may influence a wide variety of downstream genes that incorporate many crucial pathways (32). STAT6 controls the expression of several target genes, which participate in a wide range of biological and molecular processes, as was previously mentioned. MAP2K7, the high-degree gene in the pathway network, encodes MKK7, a JNK signaling pathway regulator. This gene is implicated in several cellular processes and is connected to cancer, neurological illnesses, and inflammation. Understanding the MAP2K7 gene and its protein product may help develop new treatments for these disorders. The MAP2K7 gene, which codes for the MKK7 protein, plays an important part in the initiation, development, and progression of cancer (33). In conclusion, the MAP2K7 gene is substantially involved in CRC because it promotes tumor development, metastasis, and chemoresistance. Understanding the activities and processes of the MAP2K7 gene and the MKK7 protein that the gene produces might have significance for the development of targeted treatments as well as the discovery of prognostic indicators in CRC (34). The other high-degree gene, ADCY6, encodes the protein Adenylate cyclase 6, which functions as an enzyme in the conversion of ATP into cyclic adenosine monophosphate (cAMP). Metabolism, neurotransmission, and gene expression are only a few of the biological functions that cyclic AMP controls (35-37). Researchers have found evidence that ADCY6 expression is aberrant in tumor tissues and cancer cells. ADCY6 overexpression has been shown in a variety of malignancies, including those of the breast, prostate, and pancreas. Increased tumor development, invasion, and metastasis have all been linked to elevated ADCY6 levels. Cell proliferation, angiogenesis, and the development of invasive qualities have all been linked to cAMP signaling, which has been hypothesized to be facilitated by ADCY6 (38). Although research on ADCY6’s impact on CRC is limited, the importance of these genes cannot be denied.

## Conclusion

Our results suggest that STAT6 could affect CRC cell chemosensitivity to 5-FU by activating apoptosis. When STAT6 was suppressed by siRNA, the proliferation and metastasis capability of HT-29 cells significantly decreased. Furthermore, we found that 5-FU treatment combined with STAT6 inhibition was more efficient than 5-FU treatment alone. Combination therapy was responsible for leading to apoptosis, and the regulation of apoptosis-related genes at the mRNA level, including Bcl2/Bax and caspase9, as well as CDK4 and cyclin D1, can play a key role in terminating the cell cycle. These results suggest that the combination therapy under consideration may be an effective choice for patients who have been affected.

## Authors’ Contributions

O RF performed the majority of experiments and data analysis. S N, M A, A Y, and R D contributed to carrying out the experiments and interpreted the results. V PA, SH A, and O Z wrote the manuscript and helped with experiments. B B revised the manuscript critically for important intellectual content. B A designed and conducted the project.

## Availability of Data and Materials

All data generated in this study are included in the manuscript.

## Etical Approval

All experiments and procedures were conducted in compliance with the ethical principles of Kermanshah University of Medical Sciences, Kermanshah, Iran, and approved by the regional ethical committee for medical research.

## Consent to Participate

Not applicable.

## Consent for Publication

Not applicable

## Conflicts of Interest

We certify that none of our associations or business interests poses a conflict of interest concerning the work that has been submitted.
